# Decoding inflammatory regulation in ovarian cancer at single-cell resolution

**DOI:** 10.3389/fimmu.2025.1719669

**Published:** 2026-01-12

**Authors:** Jianghao Yu, Tingting Zhou, Shuangyu Chen, Jialin Zhu, Lu Xu, Yibo He, Wei Chen

**Affiliations:** 1 The First Affiliated Hospital of Zhejiang Chinese Medical University (Zhejiang Provincial Hospital of Chinese Medicine), Hangzhou, Zhejiang, China; 2 School of Medical Technology and Information Engineering, Zhejiang Chinese Medical University, Hangzhou, Zhejiang, China; 3 The Second Affiliated Hospital Zhejiang University School of Medicine, Hangzhou, Zhejiang, China

**Keywords:** ovarian cancer, chronic inflammation, single-cell technology, tumor microenvironment, immunotherapy

## Abstract

Ovarian cancer(OC) poses a significant clinical challenge due to its frequent peritoneal dissemination and development of chemotherapy resistance, contributing to poor patient outcomes. Chronic inflammation is a pivotal driver of disease initiation and progression. While traditional population-level studies fail to capture cellular heterogeneity, recent advances in single-cell technologies including scRNA-seq, spatial transcriptomics, and multi-omics, have enabled high-resolution analysis of inflammatory regulation within the ovarian cancer microenvironment(OCME). This review synthesizes how single-cell approaches have elucidated inflammation-driven remodeling of OCME components, immune cell signaling pathways, and dynamic inflammatory-immune interactions. Focusing on the “dual microenvironments” of ovarian cancer, we discuss mechanisms of immune suppression, stromal reprogramming, and therapy resistance, along with current progress and challenges in translating these insights into precise diagnostic, targeted therapeutic, and immunotherapeutic strategies.

## 1 Introduction

OC has the highest mortality rate among gynecological malignancies (1). Globally, over 300,000 new cases are diagnosed annually, with a 5-year survival rate of less than 50% (2). The core clinical dilemma lies in the hidden early symptoms, high incidence of peritoneal and ascites metastasis, and frequent occurrence of chemotherapy resistance (3). Epidemiological studies link chronic inflammation to OC carcinogenesis: women with endometriosis face a 2.5-fold higher OC risk and the risk rises to over 4-fold for ovarian endometriosis (4), and a history of pelvic inflammatory disease is associated with increased risk of OC (5), underscoring a close correlation between inflammation and tumorigenesis (6).

Previous studies relied on bulk sequencing, which cannot distinguish specific responses of key cell subpopulations (7)and overlooks OCME heterogeneity, while the tumor microenvironment(TME) can be a strong driver of tumor aggression (8). Additionally, traditional imaging techniques fail to capture the dynamic changes of inflammatory signals during OC peritoneal metastasis. The technical limitation has long left the molecular mechanism of inflammation regulation of OCME in a “black box”, becoming a critical bottleneck restricting OC mechanism research and clinical translation.

The advent of single-cell resolution technologies offers a breakthrough (9): Single-cell RNA-sequencing(scRNA-seq) enables high-throughput single-cell transcriptome analysis to accurately identify inflammation-responsive cell subpopulations and their gene expression profiles, as well as gene regulatory networks; Spatial transcriptomics further unravels cellular spatial distribution and interactions in OCME to reveal the regional distribution pattern of inflammatory signals; single-cell multi-omics integrates transcriptomic and proteomic analyses to address the key issue of transcriptome-function disconnect, enabling direct detection of inflammatory pathway protein activity to clarify immune cells’ functional status (10–13). Based on these technologies, researchers can track inflammatory signals regulation of OCME cellular components at the single-cell level, analyze immune cell signaling network mediated by inflammation, and capture dynamic interaction patterns between inflammation and immune cells, providing unprecedented opportunities to deepen understanding of OC pathogenesis and develop novel targeted therapies. The article will systematically review the regulatory mechanisms and clinical applications of inflammation in OCME based on the research progress of single-cell technology, aiming to offer new insights for the field of OC research.

## 2 Single-cell technology deciphers the pathological remodeling of the dual microenvironments in the OCME

### 2.1 The physiological homeostasis basis of the dual microenvironment

The OCME comprises the extracellular matrix(ECM), containing chemokines, inflammatory cytokines, integrins, matrix metalloproteinases(MMPs), and other secreted molecules; and stromal cells, including cancer cells, cancer stem cells, peritoneal mesothelial cells(PMCs), pericytes, cancer-associated fibroblasts(CAFs), endothelial cells(ECs), and immune cells (14). The TME in OC exhibits greater complexity for its interconnected signaling networks and its unique peritoneal-ascites dual microenvironment (15), which promotes drug resistance and metastatic phenotypes. Disrupted homeostasis in OCME is the core driver of OC progression.

The normal ovarian-peritoneal microvironment has the core function of maintaining homeostasis: epithelial cells, as the main origin cells of OC, are orderly arranged, maintain ovarian endocrine balance via estrogen/progesterone secretion, and produce cytokines to participate in basic immune regulation, including IL-6, IL-1 and macrophage colony-stimulating factor(M-CSF) (16); the ECM provides structural support and regulates cellur function with ovarian tumors (17–20); the concentration of inflammatory factors remain low, only briefly increasing during physiological processes like ovulation (21); macrophages sustain physiological homeostasis and innate immune response via antigen presentation, phagocytosis, TME hemostasis, and other immunomodulatory processes (22); a monolayer of PMCs forms the membrane that lines the abdominal cavity and all peritoneal organs to prevent tumor cell adhesion and invasion in the peritoneum (23).

### 2.2 Pathological remodeling features revealed by single-cell technology

After the occurrence of OC, OCME undergoes significant pathological changes, with distinct features in peritoneal and ascitic microenvironments, single-cell techniques provide a high-resolution perspective for analyzing these dynamic changes.

OC cells preferentially colonize in the milky-spotted fatty tissues within the peritoneal cavity, which contain aggregates of immune cells and the fat cells provide energy for the cancer cells, priming the peritoneum as a premetastatic niche for OC (24). The OC cells secrete the same cytokines as epithelial cells such as IL-6, IL-1 and M-CSF, while normally secreted cytokines are recruited into the dysregulated autocrine cycle that drives tumor progression (25) and induce the regulation of immunosuppressive bias by inflammatory factors in the peritoneum. In an peritoneal microenvironment rich in IL-6, IL-1 and M-CSF, monocytes polarize into M2 TAMs, which express elevated inflammatory/inhibitory cytokines such as IL-10 and TGF-β, induce tumor angiogenesis via growth factors including VEGF and PDGF, and secret anti-inflammatory cytokines that recruit Tregs, further perpetuating an immunosuppression (26)the immune cell profile established through scRNA-seq revealed that in addition to the established TAMs, there were also transcriptionally distinct macrophages present in the tumor and trajectory reconstruction conducted at the subgroup level demonstrated the differentiation process from monocytes to macrophages (24, 27). A TAM subtype that generates a large amount of BAGs has been identified, which can bind to IFITM2, leading to tumor metastasis. This TAM subtype has high levels of inflammatory related VEGFA, CXCL8 and IL1β, and the trajectory analysis revealed that the classical monocytes characterized by CD14, SEL and S100A8/9 were enriched during inflammation, the corresponding highly expressed genes during their differentiation into this TAM subtype are often associated with the positive regulation of inflammatory responses and cell migration (27). Single-cell analysis of T cell population shows exhausted T cells are preferentially enriched in OCME, which were proven to consist of exhausted CD8+T cells with high expression of exhaustion-related genes such as HAVCR2(TIM-3), LAG3, PDCD1(PD-1) and CTLA-4, and found that enrichment for IFN genes in T cells suggests persistent interferon signaling could be related to the exhaustion status of these CD8+T cells (28). Moreover, dropped tumor cells acquire suspended growth ability through epithelial-mesenchymal transition(EMT) (23), forming the inflammatory connections between tumor cells and immune cells in the ascites. The tight junctions of mesothelial cells are disrupted via mesothelial-to-mesenchymal transition(MMT), a large population of CAFs accumulates, which can derive from the PMCs through MMT (23), secreting MMPs to degrade ECM components and further exacerbate peritoneal metastasis by recruiting immunosuppressive cells via chemokines like CXCL12 (29), In the ascites microenvironment,scRNA-seq identifies multiple functional CAFs subgroups, one subgroup expresses immune-related genes, such as complement factors C1QA/B/C and CFB, chemokines CXCL1/2/10/12 and cytokines IL6 and IL10, with CXCL12 and IL-6 activating JAK/STAT signaling across cancers. Just as observed in other cancer types, such as pancreatic ductal adenocarcinoma, where “inflammatory” CAFs(iCAFs) strongly express IL6 and other cytokines and may promote tumor growth and drug resistance (30). In the ascites samples of OC, each macrophage is divided into two subgroups. One subgroup includes MHC II class, IFNGR1 and M1-related genes, while the other is driven by complement factors and M2-related genes such as AIF121 and VSIG422 (30). This confirms at the single-cell level the variation axis of macrophages driven by two genetic programs.

**Figure 1:**
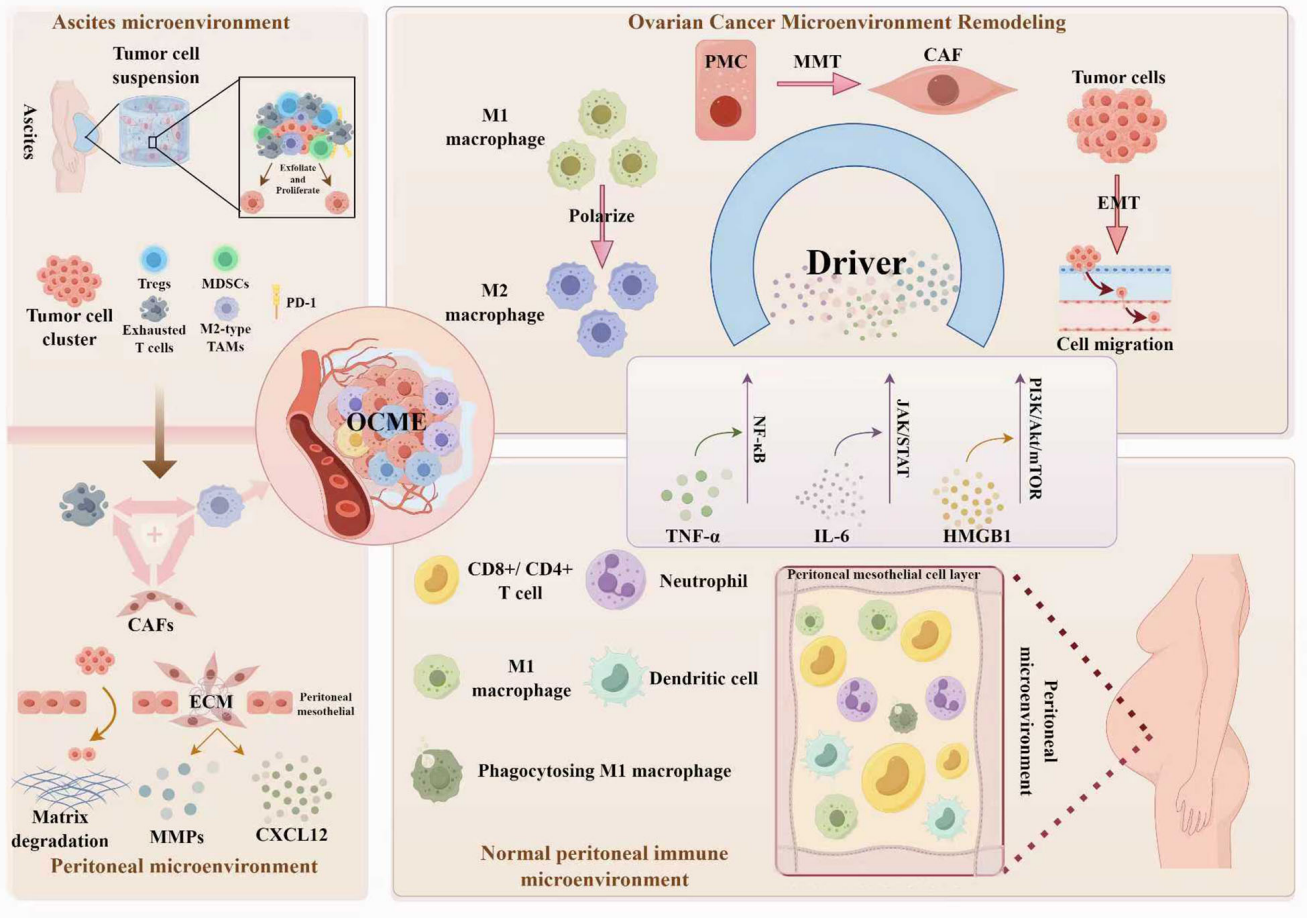
Remodeling of the dual peritoneal-ascitic aiche in ovarian cancerat single-cell resolution. Inflammation promotes the formation of a pro-metastatic dual microenvironment by orchestrating immunosuppressive ascites and matrix-remodeling peritoneal niches. **Figure 1** was created By Figdraw.

Pathological reconstruction of the dual microenvironments is a dynamic process involving multiple stages and cells, with inflammatory signals acting as a catalyst. Inflammatory factors secreted from tumor cells and microenvironmental cells accelerate CAFs activation, TAMs polarization, and immunosuppressive microenvironment formation. Conversely, pathological remodeled microenvironments further enrich inflammatory factors, creating a vicious cycle of “inflammation-reconstruction-cancer promotion” (**Figure 1**).

### 2.3 Integrate single-cell datasets and distinctive cell subpopulations

The core advantage of single-cell technology lies in revealing cell heterogeneity. In the study of OC, multiple specific cell subpopulations closely related to inflammation regulation have been identified. However, studies encompassed primary, metastatic including the peritoneum and ascites, and recurrent OC samples, the cell subpopulations exhibited extensive heterogeneity across different types of samples. Therefore, we need to perform a horizontal integration of the key single-cell datasets, further clarify the consistency and controversial points of the core findings, providing a reference for subsequent research.

Based on scRNA-seq, seven gene expression programs were defined for tumor cells in primary OC samples. Among them, cell cycle, stress, cell structure and splicing could be detected in all samples and the stress subpopulation had a relatively higher resistance score. RNA velocity and pseudo-time analysis of primary OC identified that CYR61 was highly expressed at the beginning of recurrence (31). It is considered a potential biomarker for the tumor inflammatory response in the OCME. As a pro-inflammatory factor, the level of cyr61 in ascites increases is associated with FIGO stage, initial tumor size > 10 cm, and residual tumor size, and may participate in the tumor metastasis and progression process by generating IL-6 and CRP in the OC inflammatory microenvironment (32). Another study identified the expression programs of malignant cells with coherent co-varying gene expression in OC primary tumors, ascites, and the exotic plant species from the source of ascites. Apart from the strong overlap in the cell cycle module, all samples shared three programs mainly consisting of immune or inflammatory-related genes: the inflammatory cytokine module such as IL6, TNF, IL8, IL32, the MHC II class antigen presentation module such as CD74, HLA-DRA, and the interferon response module such as IFI6, IFIT1, ISG15. These programs may be located downstream of the JAK/STAT pathway (30). All studies have confirmed the existence of LAG+PDCD+CD8+T exhausted T cells (24, 27, 28). The pseudo-time trajectory developmental analysis based on scRNA-seq depicts the gradual evolution of the immature CD4+IL7R from the root, transforming into CD8 GZMK and CD8 GZMH T cells with higher cytotoxicity, presenting a binary branching structure. One side is the terminal of exhausted T cells, and the other side is the terminal of cytotoxic T cells. In metastatic tumors, the proportion of CD8+ cytotoxic T cells is higher than that in the primary tumors. Conversely, in the primary tumors, the percentage of exhausted CD8+ T cells is higher than that in the metastatic tumors (27). All studies have confirmed the presence of M1 (CD68) and M2 (CD163/CCL18,GPNMB) cells (24, 27, 30). More importantly, they have revealed that in addition to the established TAMs, there are transcriptionally distinct macrophages in tumors. An NR1H2 cluster expressing IRF8 was identified on the metastatic tumor samples isolated from the peritoneum. This cluster is closely related to TAM. NR1H2 inhibits inflammatory genes in macrophages, and IRF8 is induced in the presence of IFNG and promotes the formation of autophagosomes (24).

A study classified CAF(THY1) into two subgroups after conducting scRNA-seq on eight samples including primary, recurrent and metastatic ones. The marker genes of CAF2 were enriched in ECM and cell adhesion functions, while the marker genes of CAF1 were enriched in response stimulation, angiogenesis and anti-apoptosis functions. It was found that the proportion of CAF1 in metastatic tumors was higher than that in primary tumors, and it was the main source of tumor cell EMT (31). The classification of the CAF subgroups of this study is different from that of other studies, the core controversy lies in whether to regard inflammatory factors as the primary criterion for classification. One study did not limit its scope to THY+ CAF, but included all fibroblasts, revealing differences between metastatic samples and primary OC samples. Fibroblasts involved in inflammatory pathways include TNFA signaling via NF-κB (33) and inflammatory responses were enriched in primary samples, whereas CAFs more closely associated with pathways sustaining tumor growth represent the most abundant subtype in metastatic OC. It was also combined with the previous studies on normal fallopian tubes(nFT), and it was found that fibroblasts shifted from a normal hormone response to an inflammatory defense/pro-tumor phenotype. The fibroblasts derived from the primary samples exhibited enriched functions related to the IFN-α/γresponse (27), indicating that they were more likely to be activated by inflammatory signals in the TME. This is consistent with the previously mentioned functions of CXCL12+IL-6+ iCAFs: by enhancing inflammatory-related signaling pathways, it became an inflammatory facilitator for tumor progression (30). Another study focused on paired primary and metastatic OC samples, dividing all fibroblasts into 5 subclusters. These subclusters included subgroups with tumor epithelial markers and EMT markers that exhibited potential for tumor-like invasion, as well as subgroups with highly expressed genes that were enriched in immune response-related pathways (34). This can also explain the above content.

## 3 Single-cell dissection of the ovarian cancer-specific inflammatory-immune regulatory network

In OCME, inflammation orchestrates immune cell functional states via activating specific signaling pathways. Aberrant activation of these pathways represents both a core mechanism of inflammation-mediated immunosuppression and a potential OC therapeutic target. Single-cell pathway analysis has uncovered multiple OC-specific inflammatory-immune signaling pathways, with divergent effects on distinct immune cell types.

### 3.1 Classical inflammatory-immune signaling pathways from a single-cell perspective

The NF-κB signaling pathway plays a key role in OC. Inflammatory factors and damage-associated molecular patterns(DAMPs) activate this pathway, increasing expression of TNF-α, IL-6, and other pro-inflammatory mediators (35) to amplify inflammation and sustain a tumor-promoting environment. Mechanistically, NF-κB primes the NLRP3 inflammasome, promotes IL-1β maturation, recruits MDSCs and TAMs, suppresses NK cell activity, and inhibits IFN-γ-mediated antitumor responses (36). Notably, NF-κB has self-limiting mechanisms: it upregulates p62, which clears damaged mitochondria via mitophagy to limit excessive NLRP3 activation and IL-1β release (37). This dual role explains why NF-κB inhibition alone often fails to achieve lasting therapeutic effects. The JAK-STAT pathway exhibits cell-specific activation in OCME, linking inflammation to immunosuppression. Based on scRNA-seq, the OC cell subgroups share three highly expressed immune-related pathways that may be located downstream of the JAK/STAT pathway; The cells in ascites, such as iCAF, are highly expressed genes that secrete ligands including IL-6 and CXCL12 that activate the JAK/STAT pathway; Moreover, the analysis of a large number of signaling genes clearly indicates that the components of the JAK/STAT pathway are expressed at particularly high levels and are widespread in both malignant and non-malignant cells (30). At the level of immune cell function regulation, sustained STAT3 activation induces CD8+ T cell exhaustion, marked by PD-1 upregulation (38). It polarizes macrophages toward M2 phenotype and restrains excessive Th1 responses (39, 40). Collectively, these changes foster immune tolerance and disease progression.

Additional pathways contribute to OC inflammation and immunosuppression: The PI3K/Akt/mTOR pathway is activated by inflammatory signals and drives M2 polarization, T cell exhaustion, and chemotherapy resistance (41–43)Wnt/β-catenin and MAPK/ERK signaling promote tumor proliferation, invasion, and immune evasion (44), while Notch signaling facilitates NLRP3 activation and Treg differentiation (45, 46).

### 3.2 Single-cell dissection of novel regulatory pathways in the ovarian cancer inflammatory-immune network

Conventional bulk sequencing fails to capture cellular heterogeneity, making it difficult to resolve cell type-specific signaling pathway activation patterns within the TME. In contrast, single-cell technologies have enabled the identification of multiple novel inflammatory-immune regulatory pathways in OC. These pathways, which exert precise control over immune cell functional states and intercellular communication, constitute a core mechanism of inflammation-mediated immunosuppression and offer potential new targets for targeted therapies.

The discovery of the CXCL12–CXCR4 axis by scRNA-seq highlights the power of single-cell resolution in ovarian cancer research. In high-stromal HGSOC, CXCL12 is specifically overexpressed by CA-MSCs and certain cancer cells, while its receptor CXCR4 is upregulated in NK and CD8+ T cells—a pattern masked in bulk sequencing. This ligand–receptor interaction promotes immunosuppression by sequestering CD8+ T cells in the stroma and directly impairing NK and T cell cytotoxicity, evidenced by reduced GZMB and IFN-γ secretion. Functionally, CXCR4 blockade enhances CD8+ T cell cytotoxicity over 2.3-fold. Clinically, high CXCL12–CXCR4 expression correlates with poor overall survival and diminished response to immune checkpoint inhibitors, underscoring its role in driving immunotherapy resistance (47).

The NECTIN2–TIGIT axis represents another paradigm uncovered by single-cell analysis. NECTIN2 exhibits pan-cellular expression across cancer, stromal, and myeloid cells, while TIGIT is specifically enriched in CD8+ exhausted T cells and Tregs—a heterogeneity invisible to bulk sequencing. Functioning as a broad immune checkpoint in ovarian cancer, NECTIN2–TIGIT engagement dampens TCR signaling, exacerbates T cell exhaustion marked by elevated PDCD1 and LAG3, and enhances Treg suppression. CRISPR-mediated NECTIN2 knockout restored T cell function, doubling proliferation and increasing IFN-γ secretion 1.8-fold. Clinically, high NECTIN2 correlates with shorter progression-free survival, upregulated expression in advanced IC2 stage, and accumulation of exhausted CD8+ T cells, highlighting its role in late-stage immunosuppression (48).

In addition to the aforementioned pathways, we have summarized several other key communication axes in **Table 1**.

**Table 1:** Emerging inflammatory-immune signaling pathways in ovarian cancer revealed by single-cell analyses and their therapeutic implications.

Signal pathway	Key molecules	Core function	Clinical significance	References
mTOR-eIF2α-ATF4-COL1A1	mTOR, eIF2α, ATF4, COL1A1	Drives fibrotic barrier formation and M2 macrophage polarization.	Links high stromal activity to chemotherapy resistance and poor survival.	(78)
MHC-II	HLA-DRA, CD74, CXCL14	Activates CD4+ T-cells; may can transform into immunosuppressive myCAFs.	Represents a double-edged sword in anti-tumor immunity.	(48)
cGAS-STING	cGAS, STING, TBK1, IRF3, Type I IFN	Context-dependent: promotes anti-tumor immunity or stromal-driven immunosuppression.	A key prognostic marker and therapeutic target for combination therapies.	(79)
SPP1-CD44	SPP1, CD44	SPP1+ TAMs secrete SPP1 to bind CD44 on CD8+ T cells, inducing exhaustion and impairing anti-tumor function.	Poor prognosis marker; axis blockade restores T-cell function and inhibits tumor growth.	(80)
CLIC3-Integrin β1-PI3K-AKT	CLIC3, ITGB1, PI3K, AKT	Enhances chemotherapy resistance and cell survival.	CLIC3 high expression indicates poor prognosis and chemoresistance.	(81)
JAG2-Notch1-RBPJ-eTreg	JAG2, Notch1, RBPJ, FOXP3	Drives immunosuppressive Treg differentiation from CD4+ T-cells.	Correlates with poor prognosis and anti-PD-1 resistance; targetable.	(45)

### 3.3 Single-cell profiling of inflammatory and immune crosstalk

Single-cell co-expression analyses unveil a core signaling network wherein NF-κB, JAK/STAT, and PI3K/Akt/mTOR pathways engage in coordinated crosstalk. A positive feedback circuit exists between NF-κB and JAK/STAT: NF-κB-driven IL-6 production activates JAK/STAT, which reciprocally enhances NF-κB via p300-mediated acetylation, collectively amplifying inflammatory and immune polarization (49). Simultaneously, PI3K/Akt/mTOR synergizes with NF-κB by promoting IκB kinase phosphorylation and suppressing apoptosis, thereby fine-tuning immune responses, tumor metabolism, and ultimately influencing therapeutic efficacy (49, 50).

Building upon this foundation single-cell resolution analyses have further uncovered multiple emerging pathways that engage in intricate crosstalk with core networks to collectively shape the immunosuppressive microenvironment. For instance, chronic activation of the cGAS-STING pathway in stromal cells drives inflammation and promotes T cell exhaustion through mediators such as type I interferons, acting synergistically with NF-κB (51). Meanwhile, JAG2-Notch signaling facilitates Treg differentiation via tumor-associated neutrophils, complementing the STAT3-mediated suppression of T cell function (45). These pathway interactions, revealed at single-cell resolution, collectively constitute an ovarian cancer-specific inflammatory-immune regulatory network with profound implications for therapeutic efficacy.

Signaling pathways form a functionally integrated tumor-promoting network through multi-layered crosstalk. For example, Midkine(MDK) integrates upstream microenvironmental signals including hypoxia and estrogen, and via its diverse receptor array comprising ALK and LRP1, converges signaling into core hubs such as PI3K/AKT, NF-κB, and STAT3. These pathways do not operate in isolation; rather, they engage in multi-level cross-communication—exemplified by Akt-mediated activation of NF-κB and transcriptional synergy between STAT3 and NF-κB—to collectively execute a unified oncogenic program. This program simultaneously activates inflammatory responses, stem-like properties, and survival mechanisms, thereby orchestrating key malignant phenotypes including immunosuppression, therapy resistance, and tumor recurrence (52).

## 4 Spatiotemporal dynamics and heterogeneity of the OCME from a single-cell perspective

Traditional analyses of the OCME, based on histology and bulk omics, have identified general immune infiltration patterns and categorized them as “inflamed” or “non-inflamed” (53). These methods detect overall shifts in major immune populations—such as T cells and macrophages—and link markers like Tregs or M2 macrophages to poor prognosis. However, they obscure critical heterogeneity by averaging cellular signals across the tumor. The inflammatory-immune interactions in OCME exhibit dynamic changes across disease stages and spatial regions. Single-cell and spatial transcriptomics decode these spatiotemporal features, providing new insights into OC progression and therapy resistance (54).

### 4.1 In the temporal dimension

A number of studies have used methods such as RNA velocity and pseudo-time reconstruction to conduct single-cell level time-resolved analyses of the OCME to infer the progression path of OC and establish dynamic gene expression maps during the tumor development process. And it was consistently observed that the number of metastatic epithelial cells increased along the trajectory in the later stage, while genes related to the immune response were significantly reduced, on a macro level (27, 34).

During early-stage OC, the OCME lacks significant immunosuppression and is characterized by predominant inflammation-initiated anti-tumor immunity. Low-level inflammatory factors secreted by OC cells recruit dendritic cells(DCs) into OCME and stimulate their maturation (55), DCs synthesize high levels of IL-12 to enhance NK cells and B/T cells immunity (56–58) and upregulate co-stimulatory molecules including LFA-3/CD58, ICAM-1/CD54, B7-2/CD86 to enhance adhesion and signal transduction (59, 60); Mature DCs migrate to lymphoid tissue, where they recruit T and B cells via chemokine release and sustain the viability of recirculating T lymphocytes (61). However, as tumors proliferate and breach local tissue barriers, leading to peritoneal metastasis and ascites formation, the dual microenvironment remodeling is completed, marking the transition to advanced disease (62). And the OCME enters a robust immunosuppressive state. Advanced OC cells secrete abundant inflammatory factors like IL-6, IL-10, TGF-β, IL-10 impairs DCs maturation, reducing co-stimulatory molecule expression and abrogating T cell activation (55); IL-6 activates the JAK/STAT pathway, inducing monocytes to differentiate into M2-TAMs, whose secreted IL-10/TGF-β further suppresses CD8+T cell cytotoxicity. Meanwhile, TNF-α activates CAFs through the NF-κB pathway and secretes CXCL12 to recruit MDSCs. Long term chronic inflammation induces the transformation of CD8+T cells from the cytotoxic subtype to the exhausted subtype(HAVCR2+LAG3+PD-1+) (63), as well the immunosuppression in OCME. Although chemotherapy kills some tumor cells, it also induces a drastic remodeling of the OCME. On the one hand, chemotherapy induces apoptosis of tumor cells, releasing a large amount of DAMPs, which abnormally activate the NF-κB pathway and enhance the PI3K/Akt/mTOR pathway (64)to promote OC chemotherapy resistance. On the other hand, a single-cell study of 15 OC patients revealed that neoadjuvant chemotherapy expands CD8+ T cells but concurrently induces immunosuppressive Myelonets that drive T cell exhaustion via NECTIN2-TIGIT signaling (65). It should be noted that the progression of tumor cells after chemotherapy-induced dormancy can evade immune surveillance (31). While the initiating cells in recurrent tumors were mainly related to the cell cycle such as MKI67, CKS2, ANLN, CDC20, CDCA8, CENPF (31), inflammatory signals no longer drive anti-tumor immunity. And single-cell analysis revealed that at OC recurrence, HRD and HRP tumors diverge: HRD tumors maintain the CD8+T cell-DC interaction niche to maintain immunogenicity, secret prostanoid E_2_ (PGE_2_) through the COX/PGE_2_ pathway to inhibit T cell proliferation and function and achieve immune evasion, whereas HRP tumors lose the CD8+T cell-DC interaction niche and accumulate TREM2+/ApoE+ TAMs (66).HRD and HRP recurrent OC have completely different immune escape mechanisms, and therefore require targeted therapeutic approaches.

### 4.2 In the spatial dimension

Spatial Transcriptomics(ST) revealed that OC and its microenvironment exhibit significant spatial heterogeneity. For instance, by integrating ST with copy number variation(CNV) analysis, a study constructed clonal evolution trees within a spatial context, visually demonstrating that OC arise from multiple CNV events, the same ancestral clone can be dispersed in different spatial regions, and the similarity of pathway activity is related to the spatial location of the clones, rather than the CNV pattern. They spatially validated the demarcation of tumor regions, characterized by high expression of epithelial markers, and stromal regions, marked by elevated fibroblast markers and revealed distinct pathway activities specific to different spatial niches: tumor regions were enriched for cell proliferation pathways such as E2F and MYC targets, whereas stromal regions were enriched for inflammatory response and EMT (67). Similarly, four immune phenotypes were constructed based on the distribution density of CD8+T cells in the tumor and stroma: the purely inflamed phenotype with CD8+ T cell enrichment in over 70% of tissue regions correlates with the highest 5-year survival rate; the mixed inflamed phenotype with enrichment in 50% to 70% of regions is associated with favorable prognosis; the excluded phenotype with scarce CD8+ T cells in under 50% of tumor regions but enriched in over 10% of stromal regions corresponds to intermediate prognosis; and the desert phenotype with sparse CD8+ T cells in under 10% of both tumor and stromal regions shows the worst prognosis.This immune phenotype classifier significantly correlated with clinical outcome, as patients with purely and mixed-inflamed phenotypes had a statistically significant longer OS compared to those with excluded and desert tumors (66). Together, these multi-faceted approaches unveil the highly organized and complex spatial and functional landscape of the OCME.

Further research has shown that the inflammatory-immune regulation in the OCME exhibits significant regional specificity, forming unique spatial functional niches at the tumor-stroma interface(TSI) and the ascites-peritoneum junction. After chemotherapy, TSI exhibits maximal cellular activity, with Myelonets differing in size and composition (65, 66). The infiltrating CD8+ T cells and IBA1+ macrophages were co-localized here, the T cell exhaustion pathway and the M1 polarization pathway were significantly enriched, and CD8+ GZMK+ T cell interactions with M2 macrophages at TSI predict poor survival (65, 68). At the ascites-peritoneum junction, ascites constructs the pre-metastatic microenvironment through two mechanisms: one is to disrupt the integrity of the mesothelial barrier, and the other is to regulate the behavior of ECM and tumor spheres, promoting the peritoneal migration of OC cells. Moreover, within ascites, tumor cells, mesothelial-derived CAFs, and infiltrating leukocytes produce a multitude of factors, including but not limited to cytokines, chemokines, and growth factors. These autocrine and paracrine soluble molecules form complex signaling networks that govern, in part, tumor-peritoneum interactions (69).

These findings inform clinical translation. For example, targeted depletion of M2 TAMs to restore CD8+T cell cytotoxicity in advanced OC (70); inhibiting the IL-6 pathway to attenuate STAT3-mediated tumor growth promotion (71); and modulating DCs migration to increase intratumoral distribution, which may enhance anti-tumor immunity.

## 5 Single-cell technologies guiding clinical translation of targeted therapies for OC

Based on the single-cell analysis of the OCME cell heterogeneity, inflammatory-immune regulatory pathways, and their spatiotemporal dynamic characteristics, a number of molecular targets and cell subpopulation markers with clinical translational value have been identified, providing a basis for targeted treatment of OC.

It is known that CD163+M2 TAMs are key immunosuppressive cells in advanced OC. However, a single-cell study revealed that one IRF8+NR1H2 macrophage cluster and another CD274 cluster exhibited similar gene expression and were similar to M1 macrophages during the anti-tumor immune response of early-stage OC, and IRF8 and CD274 were upregulated in an activation-dependent manner. Perhaps the macrophages in the NR1H2+IRF8+ cluster are in a transitional state, stimulating them to become M1 macrophages, conversely, preventing them from becoming M2 TAMs, might be a method of OC immunotherapy (24). The immunosuppressive phase of OC is characterized by abnormal activation of inflammation-driven signaling pathways. Various JAK/STAT inhibitors have been proven to have anti-tumor properties in OC cell lines. Among them, the natural compound α-Hederin functions as a dual JAK1/2 inhibitor, with microscale thermophoresis analysis confirming its high binding affinity for both JAK1 and JAK2. This direct interaction effectively suppresses STAT3 phosphorylation and nuclear translocation, leading to inhibition of tumor proliferation, migration, and EMT. Furthermore, α-Hederin exhibits synergistic effects with cisplatin in overcoming platinum resistance (72). Additionally, Wnt/β-catenin inhibitors target pathway dysregulation associated with tumor stemness and chemoresistance. Preclinical evidence confirms that suppression of this signaling cascade reduces tumor growth while enhancing sensitivity to conventional therapies, supporting their integration into combination treatment regimens (73). During the OC progression process, the signaling pathways related to energy metabolism, such as cell cycle and tumor cell proliferation, have significantly strengthened. PARP inhibitors, particularly niraparib, have shown significant clinical efficacy in heavily pretreated patients. The QUADRA trial demonstrated a 28% objective response rate in HRD-positive, platinum-sensitive populations, with substantial improvements in both duration of response and overall survival (73). After chemotherapy, the NECTIN2-TIGIT signaling pathway is significantly activated between myeloid cells and CD8+T cells, and it is a key driver of T cell exhaustion. In patient-derived immunocompetent cultures(iPDCs), monotherapy with anti-TIGIT or combination therapy with anti-PD-1(pembrolizumab) can significantly enhance the expression of granzyme B and IFN-γ in CD8+T cells in samples after chemotherapy, activating the anti-tumor function of T cells, and the treatment response is more significant in samples with high expression of NECTIN2-TIGIT (65). This method may reverse the T-cell exhaustion state in HRD-recurring OC that maintains immunogenicity.

## 6 Current challenges and future directions

Single-cell technologies are introducing a new dimension to the clinical management of OC, extending beyond conventional histological subtyping and genotyping. For instance, the analysis of circulating cells from ascites or peripheral blood enables the non-invasive construction of a “real-time” TME atlas, thereby allowing dynamic monitoring of therapeutic response and the evolution of drug resistance. In the future, clinical trial design will no longer rely solely on tumor histology or a limited set of driver gene alterations. Instead, it will integrate single-cell–resolved “cellular subset signatures”—such as specific tumor-associated macrophage lineages or T cell state ratios—to define molecular subtypes, predict treatment outcomes, and refine patient stratification. Realizing this vision may require a new paradigm capable of systematically integrating and reasoning over these complex features, as demonstrated by the potential of graph-enhanced AI knowledge graphs like RSA-KG in integrating multimodal data to enhance clinical decision-making and ultimately paving the way for authentic precision immunotherapies (74).

However, OC research faces two key challenges: significant TME spatiotemporal heterogeneity hindering precise targeting (66), and extensive compensatory pathways between inflammation and immunosuppression leading to treatment resistance (75). Future efforts should use single-cell and spatial transcriptomics to map regional molecular targets, guiding rational drug combinations. Developing organoids mimicking human tumor environments (76), combined with smart delivery systems, to improve preclinical predictions (77). We could design synergistic therapies targeting specific immune cells/pathways and validate them through well-designed clinical trials to advance personalized treatment.

## 7 Conclusion

Breakthroughs in single-cell and spatial omics technologies have elucidated the pivotal role of inflammatory and immunosuppressive networks within the OCME. Chronic inflammation, mediated by key signaling pathways including NF-κB, JAK/STAT, and PI3K/Akt/mTOR, dynamically remodels the cellular composition and function of the OCME, driving immune suppression, stromal remodeling, and tumor metastasis. Single-cell resolution uncovers heterogeneous cellular responses and interactions under inflammatory signals, as well as the spatiotemporal dynamics of chemotherapy resistance and immune evasion. These findings provide precise molecular targets for developing novel therapies directed at the inflammation-immunity axis. They propel OC treatment toward combination targeted and immunotherapy, along with personalized approaches based on microenvironment molecular subtyping, offering new prospects for improving patient prognosis.
